# A survey exploring biomedical editors’ perceptions of editorial interventions to improve adherence to reporting guidelines

**DOI:** 10.12688/f1000research.20556.3

**Published:** 2019-12-23

**Authors:** David Blanco, Darko Hren, Jamie J. Kirkham, Erik Cobo, Sara Schroter

**Affiliations:** 1 Statistics and Operations Research Department, Universitat Politècnica de Catalunya, Barcelona, Spain; 2 Université de Paris, CRESS, INSERM, INRA, Paris, France; 3 Faculty of Humanities and Social Sciences, University of Split, Split, Croatia; 4 Centre for Biostatistics, Manchester Academic Health Science Centre, University of Manchester, Manchester, UK; 5 The BMJ, London, UK

**Keywords:** Completeness of reporting, Journal policies, Quality of reporting, Reporting guidelines, Survey, Barriers, Facilitators

## Abstract

**Background: **Improving the completeness of reporting of biomedical research is essential for improving its usability. For this reason, hundreds of reporting guidelines have been created in the last few decades but adherence to these remains suboptimal. This survey aims to inform future evaluations of interventions to improve adherence to reporting guidelines. In particular, it gathers editors’ perceptions of a range of interventions at various stages in the editorial process.

**Methods: **We surveyed biomedical journal editors that were knowledgeable about this topic. The questionnaire included open and closed questions that explored (i) the current practice of their journals, (ii) their perceptions of the ease of implementation of different interventions and the potential effectiveness of these at improving adherence to reporting guidelines, (iii) the barriers and facilitators associated with these interventions, and (iv) suggestions for future interventions and incentives.

**Results: **Of the 99 editors invited, 24 (24%) completed the survey. Involving trained editors or administrative staff was deemed the potentially most effective intervention but, at the same time, it was considered moderately difficult to implement due to logistic and resource issues. Participants believed that checking adherence to guidelines goes beyond the role of peer reviewers and were concerned that the quality of peer review could be compromised. Reviewers are generally not expected to focus on reporting issues but on providing an expert view on the importance, novelty, and relevance of the manuscript. Journals incentivising adherence, and publishers and medical institutions encouraging journals to take action to boost adherence were two recurrent themes.

**Conclusions: **Biomedical journal editors generally believed that engaging trained professionals would be the most effective, yet resource intensive, editorial intervention. Also, they thought that peer reviewers should not be asked to check RGs. Future evaluations of interventions can take into account the barriers, facilitators, and incentives described in this survey.

## Abbreviations

RGs: reporting guidelines; CONSORT: Consolidated Standards of Reporting Trials; RCT: Randomised controlled trials; EQUATOR: Enhancing the QUAlity and Transparency Of Health Research; MiRoR: Methods in Research on Research; STROBE: STrengthening the Reporting of OBservational studies in Epidemiology; PRISMA: Preferred Reporting Items for Systematic Reviews and Meta-Analyses; APCs: article processing charges; CME: continuing medical education; ICJME: International Committee of Medical Journal Editors.

## Introduction

Transparent and accurate reporting of research is essential for increasing the usability of available research evidence. Reporting guidelines (RGs) can be useful tools to help authors report research methods and findings in a way that they can be understood by readers, replicated by researchers, used by health care professionals to make clinical decisions, and included in systematic reviews ^1^. Since the inception in 1996 of the Consolidated Standards of Reporting Trials (CONSORT) for the reporting of randomised controlled trials (RCTs) ^2^, more than 400 RGs for different study types, data, and clinical areas have been developed. These RGs can be found in the library of the Enhancing the Quality and Transparency Of Health Research (EQUATOR) Network ^3^.

Biomedical authors’ adherence to RGs has been observed to be suboptimal ^4^. Consequently, in recent years various stakeholders have proposed, and sometimes evaluated, the impact of different types of interventions to improve this adherence. These interventions were identified and classified in a recently published scoping review ^5^. We found that the strategies most widely used by journals have been shown not to have the desired effect ^6– 9^ and this highlighted the need for the implementation and evaluation of the other interventions proposed ^5^.

This paper reports a survey aimed to inform the future evaluation of interventions to improve adherence to RGs. In particular, we focused on interventions that can be implemented at various points in the editorial process. Our specific objectives were to explore the perceived ease of implementation of different interventions and the potential effectiveness of these at improving adherence to RGs; to map the barriers and facilitators associated with these interventions; to determine possible solutions to overcome the barriers described, and to identify further editorial interventions that could be implemented and subsequently evaluated.

## Methods

### Participants

Purposive sampling was used to recruit biomedical editors that were expected to be knowledgeable and experienced in the topic we aimed to explore. We recruited participants not based on their representativeness of all medical journals but on the fact that they were “information-rich cases” ^10^.

Participants were sampled from three sources: (i) editors of journals that had published studies describing interventions to improve adherence to RGs identified in our scoping review ^5^, (ii) members of the Methods in Research on Research (MiRoR) Network with current editorial positions and (iii) editors of the top-10 journals (based on impact factor) of BMJ Publishing Group which, apart from being one of the partner institutions of MiRoR, has published the main RGs ^2, 11– 13^) and has traditionally performed research to improve the transparency and quality of biomedical publications ^14^. The authors of this survey who met the eligibility criteria were excluded as potential participants.

### Recruitment

The survey was only open to editors that we invited to participate. We contacted three editors (including the editors-in-chief) of each of the sampled journals, as well as individual editors from the group (ii) above. By replying to our invitation email, participants could suggest further editors that they considered could contribute to the survey. To contact editors not known to us we sought email addresses in the public domain. The survey was not advertised on any website.

### Survey administration

The survey was administered by SurveyMonkey ^15^ and was open between 27 November 2018 and 24 February 2019. Participants were sent a personalised email inviting them to complete an online survey investigating their opinions about different editorial interventions to improve author adherence to RGs. Each invitation was tied to a unique email address. Two reminders to complete the survey were sent to non-responders at four and eight weeks after the initial mailing.

Participants could edit their responses while completing the survey. However, they could not re-enter the survey once it was completed as no two entries from the same IP address were allowed. We did not offer any incentives for completing the survey.

### Response rates

We recorded the view rate of the invitation email (subjects opening the invitation email/subjects invited), the response rate (subjects completing the survey/subjects invited), and the completion rate (subjects completing the survey/subjects completing the first question of the survey).

### Questionnaire development

Our previous scoping review ^5^ identified 31 interventions targeting different stakeholders in the research process. For use in this survey we chose a smaller subset of nine interventions that could be implemented during the editorial process as our focus was on journal editors’ perceptions (see Box 1).

Box 1. **Interventions included and their targets**

**A**. Interventions targeting authors:
  • A requirement for authors to submit a completed RG checklist (using all appropriate extensions, if applicable) indicating the page numbers where each item is addressed ( **Intervention 1**)
  • A requirement for authors to submit a populated RG checklist with text from their manuscript in order to facilitate the peer review process ( **Intervention 2**)
  • A requirement for authors to highlight in the manuscript where each RG item is addressed ( **Intervention 3**)
  • A requirement for authors to include new subheadings within their manuscript corresponding to different RG items within the traditional IMRaD format (Introduction, Methods, Results, and Discussion) ( **Intervention 4**)
  • A requirement for authors on submission to use a freely available writing aid tool that guides authors through the RG checklist items, shows the key elements that need to be reported, and includes examples of adequate reporting (e.g. COBWEB) ( **Intervention 5**)
**B**. Interventions targeting peer reviewers:
  • Instruct peer reviewers to use the appropriate RGs when assessing a manuscript ( **Intervention 6**)
  • Instruct peer reviewers to scrutinise the completed RG checklist submitted by the authors and check its consistency with the information reported in the manuscript ( **Intervention 7**)
**C**. Interventions targeting editorial staff:
  • An evaluation of the completeness of reporting by a trained editor (or editorial assistant), who would return incomplete manuscripts to authors before considering the manuscript for publication ( **Intervention 8**)
**D**. Interventions targeting authors, peer reviewers, and editors:
  • Training for authors, peer reviewers, and editors on the importance, content, and use of RGs (e.g. The EQUATOR Network toolkits) ( **Intervention 9**)

The survey combined open and closed response questions to seek participants’ perceptions of a series of interventions to improve authors’ adherence to RGs that could potentially be implemented during the editorial process. We pilot tested the draft survey questionnaire with two collaborators of the MiRoR project who currently hold editorial positions. They were asked to review the survey for its clarity and completeness and to provide suggestions on how to improve its structure.

Based on feedback from the pilot we decided not to include the intervention “Implementation of the automatic tool Statreviewer ^16^” since participants were not aware of this software and stated that their perceptions would strongly depend on details about how it operates which are not publicly available.

The survey combined open and closed response questions to seek participants’ perceptions of a series of interventions to improve authors’ adherence to RGs that could potentially be implemented during the editorial process. We structured the questionnaire (see Figure S1, *Extended data* ^17^) as follows:

- *Part 1: Current practice*. Participants were asked to describe the measures their journal currently takes to improve adherence to RGs.
- *Part 2: Perceptions of nine potential interventions*. Participants were asked to indicate on 5-point Likert scales (i) how easy it would be (or was) to implement these interventions at their journals (1-very difficult, 2-moderately difficult, 3-neither difficult nor easy, 4-moderately easy, 5-very easy) and (ii) how effective they thought the interventions would be (or was) at improving adherence to RGs if these were implemented at their journals (1-very ineffective, 2-moderately ineffective, 3-neither ineffective nor effective, 4-moderately effective, 5-very effective). We included images to clarify meanings and context to prompt participants to think about the benefits and drawbacks of the interventions. Free text boxes were included so participants could justify their responses.
- *Part 3: Identifying the barriers and facilitators.* Participants were asked to choose which intervention they considered potentially the most effective for their journal at improving adherence to RGs. They were asked to describe (i) why they thought that intervention would be the most effective, (ii) what the main difficulties in implementing that intervention would be, and (iii) how they would try to overcome these difficulties.
- *Part 4: Further interventions.* Participants were asked for further suggestions of possible interventions, including modifications and combinations of the interventions previously discussed.
- *Part 5: Demographic questions*.

The survey was distributed over 18 pages with 1 to 3 items per page. These items were not randomised.

### Data analysis

For quantitative data (Part 2 of the questionnaire), we used R version 3.6.0 ^18^. As these data were ordinal, we calculated medians together and the 1st and 3rd quartiles. We excluded from the analysis one questionnaire where the participant just opened the survey and left without answering any question. We did not exclude any questionnaire based on the amount of time that the participant needed to complete it.

For qualitative information, the lead investigator (DB) used the software program NVivo 12 ^19^. We mapped the barriers and facilitators for each of the interventions explored, as well as other key themes such as the incentives for the use of RG and the implementation of further editorial strategies. The initial mapping made by the lead investigator was discussed with another investigator (SS) and subsequently refined.

For Part 1 of the survey ( *Current practice*) the unit of measure were the journals and therefore editors of the same journal were grouped. This was due to the fact that participants’ answers represented an overarching policy and not an individual’s opinion. For all other parts of the survey (Part 2 to Part 5), we analysed editors’ responses independently, no matter what their journal was.

### Ethics approval & informed consent

The Research Committee of the Governing Council of the Universitat Politècnica de Catalunya (UPC) granted ethical approval for this study (Reference EC 01, Date 2 May 2018).

In the invitation email, we informed survey participants that (i) the completion of the survey indicated consent to participate, (ii) they were free to stop and withdraw from the study at any time without providing a reason, (iii) the estimated time to complete the survey was 15 minutes, (iv) any identifiable information obtained in connection with this survey would remain confidential, and (v) the results would be submitted for publication and the anonymised dataset would be made publicly available in the Zenodo repository. The original dataset was kept in a password-protected folder in Google Drive.

### Reporting guidelines

We consulted the Checklist for Reporting of Results of Internet E-Surveys (CHERRIES) ^20^ and the Consolidated criteria for Reporting of Qualitative research (COREQ) ^21^ guidelines to produce this research report.

## Results

Of the 99 editors invited, 42 opened the invitation (view rate 42%), and 24 completed the survey (response rate 24%) from the 25 who started it (completion rate 96%). The average time spent completing the survey was 15 minutes (SD = 8.5 minutes). Among the 24 participants who completed the survey, nine (37%) worked for seven different journals that had published studies on improving adherence to RGs, seven (29%) worked for five top-10 BMJ journals, four (17%) were members of the MiRoR Network that hold editorial positions in four journals, and a further four (17%) were suggested by other participants based on their expertise on the topic and were editors of three different journals. The 20 journals represented in the survey are listed in Table 1.

**Table 1.** Journals represented in the survey.

**Journals that have published research on RGs**	Trials
	The Lancet
	PLOS ONE
	BMC Medicine
	BMC Medical Research and Methodology
	Journal of Oral Rehabilitation
	Journal of Clinical Epidemiology
**Journals that belong to BMJ top-10**	BMJ
	Archives of Disease in Childhood
	BMJ Open
	BMJ Quality & Safety
	British Journal of Sports Medicine
**MiRoR Network members’ journals**	Clinical Chemistry
	Systematic Reviews
	Research Integrity and Peer review
	Journal of Global Health
**Other journals**	F1000
	BMJ Open Science
	Scientific Reports

Participants had a variety of editorial roles (editor-in-chief, senior editor, associate editor or others). Most of them were involved in manuscript decision-making and had less than 15 years of experience as journal editors (see Table 2). The anonymised responses from all 24 participants can be accessed in Zenodo ^22^.

**Table 2.** Demographic characteristics of the 24 participants.

N=24		
**Current position**	Working full time as a journal editor	8 (33%)
	Working part time (equal or more than 0.5 of their time) as a journal editor	1 (4%)
	Working part time (less than 0.5 of their time) as a journal editor	14 (59%)
	Other (Volunteer editor)	1 (4%)
**Editorial role**	Editor-in-chief	10 (41%)
	Senior editor	4 (17%)
	Associate editor	4 (17%)
	Other (Editorial director, Technical editor, Assistant editor)	6 (25%)
**Involvement in manuscript** **decision-making**	Yes	22 (92%)
	No	2 (8%)
**Years of experience as a journal editor**	<5	8 (33%)
	5–15	12 (50%)
	15–25	3 (13%)
	>25	1 (4%)

### Current practice

Respondents worked at 19 journals. Most respondents’ journals (11/19, 58%) request authors to submit a completed RG checklist with page numbers indicating where the items are addressed when they submit their manuscript. A further seven (37%) instruct but do not request authors to do it, and one (5%) does not request or instruct authors. Among the journals requesting the submission of checklists, four (4/11, 36%) also explicitly ask peer reviewers to use the completed RGs when assessing manuscripts, one (1/11, 9%) asks peer reviewers general questions about the completeness of reporting, and one performs an evaluation of the completeness of reporting by a trained editor using RGs before the initial decision is made on the manuscript. We observed no incongruences between the answers of editors from the same journal. Some respondents mentioned that in their journals (n=4) the interventions described were only applicable to the study types corresponding to the most established RGs (CONSORT, PRISMA ^11^, or STROBE ^12^) for trials, observational studies and systematic reviews respectively.

### Perceptions of nine potential interventions

**Figure 1.**
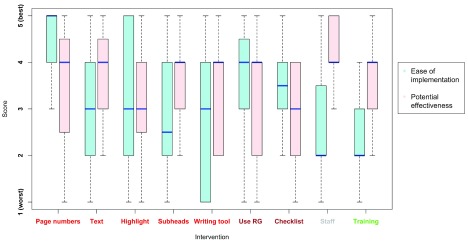
Scores for perceived ease of implementation and perceived effectiveness. Box plots show the 1 ^st^, 2 ^nd^ (medians, represented as blue horizontal lines), and 3 ^rd^ quartiles of the data. The whiskers extend up to 1.5 times the interquartile range from the top (bottom) of the box to the furthest datum within that distance. Interventions whose names are shown in red target authors, those in brown target peer reviewers, the one in grey target editors or administrative staff and the one in green targets all these stakeholders. Box 1 shows a detailed explanation of each intervention.

The mean scores for perceived ease of implementation and potential effectiveness for each intervention are shown in Figure 1.

The two most common interventions were considered the easiest ones to implement: the median scores (1 ^st^, 3 ^rd^ quartiles) for requesting authors to submit checklists with page numbers (Intervention 1) and for asking peer reviewers to use RGs (Intervention 6) were 5 (Q1: 4, Q3: 5) and 4 (Q1: 3, Q3: 5), respectively. By contrast, interventions related to training (Intervention 9), editor involvement in checking completeness of reporting (Intervention 8) and reformatting of the text based on RG requirements (Intervention 4, Intervention 5) were considered the most difficult to implement.

An evaluation of the completeness of reporting by a trained editor was considered the most effective intervention at improving adherence to RGs (Median: 4, Q1: 4, Q3: 5) and the two targeting peer reviewers (Interventions 6 and 7) were perceived as being the least effective (Median: 4, Q1: 2, Q3: 4; Median: 3, Q1: 2, Q3: 4). 

### Identifying the barriers and facilitators

This section presents the perceived barriers and facilitators of the interventions considered and editors’ suggestions for making the interventions more effective. Table S1 in *Extended data ^17^* shows a full description of these.

**A) Interventions targeting authors (1–5)**

The main barriers associated with all of the interventions targeting authors was that authors have to state their adherence to the relevant RG and this does not equate to actual compliance. Moreover, it is resource intensive for journals to check that these requirements are appropriately met by authors. Some editors highlighted that Interventions 3, 4, and 5 would involve special formatting of the submitted manuscript, which could be cumbersome for authors given that manuscripts are often submitted to multiple journals with different formats before being accepted. This is particularly relevant for journals with high rejection rates as it could cause frustration for authors. Some participants mentioned logistical issues as their journal’s manuscript tracking system is not set up to accommodate these interventions. In addition, changes in the manuscript’s format could be incompatible with the journal’s house style.

Intervention 1 was generally considered quick and straightforward for authors, but several participants indicated that there is published empirical evidence of little effectiveness if the checklist is not assessed by a trained editor or administrator ^5– 8^.

As Interventions 3, 4, and 5 force authors to tailor the manuscript to RG requirements, participants reported that these could make editors’ and peer reviewers’ jobs easier as the manuscript would be better structured. Importantly, readers would also be able to locate information more easily. Some editors pointed out that, to make these interventions effective, journals would need to provide templates to authors or to integrate these interventions in the submission system. However, some of these interventions (Interventions 2 and 5) were seen as more effective if they were implemented earlier on in the research process, prior to writing the manuscript.

**B) Interventions targeting peer reviewers (6, 7)**

Most respondents were negative about the potential effectiveness of implementing the two interventions targeting peer reviewers (Intervention 6 and 7) as they felt these would create too much additional work for reviewers. Participants were concerned that the quality of peer review could be compromised as reviewers are not expected to focus on reporting issues but on providing an expert view on the importance, novelty and relevance of the manuscript. Furthermore, peer reviewers may not know which RGs to use and, even if they do, the effectiveness would be dependent on their willingness to use RGs and their expertise in applying them. Several participants indicated that this work should be delegated to paid editorial staff.

**C) Interventions targeting editorial staff (8)**

This intervention was considered difficult to implement but potentially effective. The main facilitating factor for its successful implementation was that it is performed by a paid or trained professional, which lends credibility to the intervention, reduces the workload of unpaid peer reviewers, and avoids authors overclaiming adherence. The main barriers outlined for this intervention were (i) the budget issues the journal would need to face to train or hire additional editorial staff that could perform the evaluation, especially if the journal receives a large volume of manuscripts, (ii) the editorial delays it may cause, and the (iii) the potential inefficiency of assistant editors or administrators having to delegate decisions in case of doubt, given that sometimes assessing completeness of reporting is a subjective task.

To make this intervention more feasible for journals, editors suggested that the completeness of reporting evaluation could be performed only for manuscripts that are sent out for peer review and, it could be focused on a few core items (different for each RG) that would enable reproducibility. If this intervention was implemented in a journal that requires the submission of a completed checklist, editors could take advantage of the checklist to locate information.

**D) Interventions targeting authors, peer reviewers and editors (9)**

Training was seen as a potentially effective intervention but difficult to implement. Some participants highlighted that training with follow up sessions would be resource intensive for journals, and especially difficult to enforce. One participant mentioned that credits (such as CME credits ^23^) could be used to recognise hours of training. The fact that sometimes the editorial staff is based in different places and zones makes it crucial to consider flexible forms of training, such as online courses. As an example, the EQUATOR Network Toolkits section provides resources for authors, peer reviewers and journal editors ^24^. However, some participants emphasised that training should also be delivered by research institutions and medical centres.

### Further interventions and incentives for authors and journals

When asked about further potentially effective interventions that were not discussed in the survey, some editors mentioned StatReviewer, a reading tool that automatically assesses adherence to RGs and is currently under evaluation ^16^. Other respondents also mentioned the possibility of combining some of the interventions discussed in the survey, such as requiring the submission of checklists and trained editors assessing the responses with the information reported in the manuscript.

Moreover, several incentives for authors were listed, including (i) discounts on article processing charges (APCs) for authors that comply with RG requirements, (ii) academic institutions including RG use in the promotion and tenure files, and (iii) credits (such as CME credits ^23^) to recognise hours of training on the use of RGs. Journals could also be encouraged to implement certain interventions if (i) there is empirical evidence that these interventions actually improve the reporting quality of the papers or (ii) publishers or the International Committee of Medical Journal Editors (ICMJE) mandate these as a condition of submission to their journals. Even if some of these interventions are proven to be effective, some respondents reported that it is essential to convince publishers that improving the quality of reporting is a worthy investment to resource.

## Discussion

This survey explores biomedical journal editors’ perceptions of the practical aspects of the implementation of different interventions to improve adherence to RGs.

Several messages arise from this study. First of all, most editors agreed that the most effective way to improve adherence to RGs is for journals to involve trained editors or administrative staff. Interventions targeting these stakeholders were considered to be difficult to implement for most journals, either because of logistic or resource issues. However, improving the performance of editorial staff is critical ^25^ and has been shown to have a positive impact on completeness of reporting in the context of a dentistry journal ^26^. To make these type of interventions more feasible, journals could implement them only for manuscripts that are sent out for peer review. The editorial staff could also take advantage of the RG checklists submitted by authors, that could be automatically populated with text using specific software such as the the tool proposed by Hawwash *et al.* ^27^

Most editors considered that checking reporting issues is beyond the role of peer reviewers. Given the voluntary nature of peer review, requiring reviewers to use RGs would cause an additional workload that could compromise the overall quality of the reviews. If checking reporting issues becomes a standard exercise for peer reviewers, some editors are concerned that peer reviewers may be less likely to comment on important aspects of a manuscript, such as its novelty, clinical interest or implications. Furthermore, as finding peer reviewers is becoming increasingly difficult for editors ^28^, these requirements could make them even less willing to review papers. Additionally, some editors considered that the average peer reviewer does not have enough expertise to go over RG requirements.

We observed that the interventions perceived as potentially most effective improving adherence to RGs appear to be more difficult to implement. Conversely, the most common strategies seem to have been implemented based on their feasibility and not on their potential to improve completeness of reporting. This could be one of the reasons why they have failed to achieve the desired results ^6– 9^). Some of our respondents insisted that a key element is that journals, universities, and medical institutions find ways to incentivise author’s compliance with RGs. At the same time, the scientific community needs to find ways to convince publishers that improving the quality of reporting is a worthy investment so that publishers can encourage their journals to adopt strategies to boost completeness of reporting. A recent article indicates that implementing RGs through the editorial process may increase the number of citations to the research reported ^29^.

A common observation by the survey participants was that the effectiveness of the interventions proposed could depend on the types of articles considered. While RGs for randomised trial protocols, randomised trials or systematic reviews are more established, some others, including most RG extensions, are not well known to the stakeholders involved in the publication process. For this reason, it is important for journals to be clear in their “Instructions for Authors” on what RGs they mandate.

It is noteworthy to mention that, regardless of how checklists are implemented in the editorial process and who has to engage to make the interventions successful, the evaluation of completeness of reporting is a subjective task. This is mainly due to the fact that RGs are not originally designed as evaluation tools but as guidance for authors on how to report their research. For this reason, evaluators could sometimes have different views on whether authors are providing enough information to consider that certain RG items are adequately reported.

This study is subject to several limitations. The response rate was low (24%). However, researchers in health science have witnessed a gradual decrease in survey participation over time ^30^, especially among health professionals due to the demanding work schedules and increasing frequency of being approached for surveys ^31^. Some recent surveys in the field of peer review show even lower response rates (10–20%) among researchers, peer reviewers and readers ^32, 33^. It is also noteworthy that we took a pragmatic approach to identify relevant editors and the sample was small due to not many having conducted or published research on improving adherence to RGs. Whilst n=24 is a small number, the detailed and rich responses that we received showed a high level of engagement with the topic. Despite having the option to increase the sample size by contacting more editors at a lower level of hierarchy in the journals we targeted, we decided not to do it based on the response rate of the survey. That approach would have changed our sampling frame and we would potentially have had less experienced editors commenting. We took that decision as the purpose of the survey was to tap into the experience of those who had tried interventions or had shown interest in this area, instead of seeking a representative sample of editors.

Connected with this, we could expect survey participants to be more prone to adopt interventions than general biomedical editors. However, their experience could also make them more critical of certain strategies that appear to be more effective than they actually are. This could be the case for the intervention of requesting authors to submit checklists on manuscript submission, which has become popular among medical journals despite having little or no impact on completeness of reporting ^6– 9^). Editors with less experience of editorial strategies to improve adherence to RGs might expect authors and peer reviewers to respond to certain interventions in a different way than they would do.

We encourage researchers to perform further evaluations of interventions in collaboration with biomedical journals, such as the RCT our research team is currently undergoing ^34^. Our study aims to evaluate the effect on completeness of reporting of a trained researcher assessing during peer review the consistency between the CONSORT checklists submitted by authors and the information reported in the manuscript, and providing authors with a report indicating any inconsistencies found.

Providing high quality evidence of the effectiveness of different interventions at improving adherence to RGs and discussing how to make them less burdensome are key aspects needed to convince all stakeholders that this effort is worth it.

## Conclusions

Biomedical journal editors generally believed that engaging trained professionals in the process of checking adherence to RGs would be the most effective, yet moderately resource intensive, editorial intervention. Also, they thought that standard peer reviewers should not be asked to check RG requirements. 

Future evaluations of interventions to improve adherence to RGs can take into account the barriers, facilitators, and incentives for implementing editorial interventions that are described in this survey.

## Data availability

### Underlying data

Zenodo: Underlying data of the project “A survey exploring biomedical editors’ perceptions of editorial interventions to improve adherence to reporting guidelines”. DOI: https://doi.org/10.5281/zenodo.3407725 ^22^.

This project contains the following underlying data:

Survey dataset (Dataset including all survey responses).

### Extended data

Zenodo: Extended data of the project “A survey exploring biomedical editors’ perceptions of editorial interventions to improve adherence to reporting guidelines”. https://doi.org/10.5281/zenodo.3404002 ^17^.

This project contains the following extended data:

Figure S1: Survey questionnaire (Complete version of the survey questionnaire used in this project)

Table S1: Barriers, facilitators and possible improvements of the included interventions (Table containing the barriers, facilitators and possible improvements identified for each of the interventions explored in the survey)

Data are available under the terms of the Creative Commons Attribution 4.0 International license (CC-BY 4.0).
