# Supplementary material for: A survey exploring biomedical editors’ perceptions of editorial interventions to improve adherence to reporting guidelines
Source: F1000Res. 2019 Dec 23;8:1682. Originally published 2019 Sep 24. [Version 3] doi: 10.12688/f1000research.20556.3 (PMC6880264; doi:10.12688/f1000research.20556.3)
Supplement: Peer review history

# Peer review: Reviewer response for version 3

**DOI:** 10.5256/f1000research.24060.r57976

**Published:** 2020-01-20

**Authors:** Gerard Urrutia

**Affiliations:** 1 Iberoamerican Cochrane Center, Sant Pau Biomedical Research Institute, Barcelona, Spain

I’m happy with the revised version and my decision is to recommend approval as it is now.

I confirm that I have read this submission and believe that I have an appropriate level of expertise to confirm that it is of an acceptable scientific standard.

# Peer review: Reviewer response for version 3

**DOI:** 10.5256/f1000research.24060.r57975

**Published:** 2019-12-23

**Authors:** Patricia Logullo

**Affiliations:** 1 EQUATOR Network, University of Oxford, Oxford, UK

I am very happy with this new version of the manuscript. It is clear, well-reported, and the conclusions are there. Thank you for taking the time to improve it! Thank you also for the opportunity to review it.

I confirm that I have read this submission and believe that I have an appropriate level of expertise to confirm that it is of an acceptable scientific standard.

# Response

**Published:** 2019-12-23

**Authors:** David Blanco

**Affiliations:** Universitat Politècnica de Catalunya, Spain

Again, thanks for the level of detail of your comments and the time you took to make them. The paper is much clearer and better reported now.

# Peer review: Reviewer response for version 2

**DOI:** 10.5256/f1000research.23906.r57746

**Published:** 2019-12-16

**Authors:** Patricia Logullo

**Affiliations:** 1 EQUATOR Network, University of Oxford, Oxford, UK

**Positive points**

 The paper improved a lot from the last version. As I said before, this is a much-needed survey by MiRoR team, and I hope it gets fully published and indexed as soon as possible.

 The authors took care to respond to all the reviewers’ questions. However, there are still three points that were not adequately addressed, and I hope I can make some suggestions to help them solve these problems. I also found two additional problems that came up in this second version of the manuscript.

 From the 20 points I commented in my last report, the authors adequately responded/solved the problems in the items 1, 3-5, 7, 9-17, 19 and 20. Thank you for that. Below you can find comments on the remaining items (I used the same numbers as before to make it easier to locate them) and the two additional ones.

 2) **Sample.** The authors explained that they purposely searched for editors experienced with reporting guidelines. As much as I don’t agree with this approach, at least it was clearly stated now. 

 However, the explanation about the sample size as a study limitation still does not seem satisfactory. The authors added a comment in the discussion section attributing the sample size to a low response rate in surveys in general. But if the authors knew there was going to be a low response rate (and they cited very useful references for that), why did they stop recruiting? Why not try to approach other editors/journals? The authors should expand the explanation to respond to two questions:

- What were the constraints in increasing the sample? Why did you stop recruiting?
- Even choosing to use a “not representative sample”, can we trust that we should listen to the opinions of these 24 editors (a minority among the 99 invited) to say something is feasible or not, for example? What is the effect of this small sample size on the “take-home messages” of this study?

These points were not addressed in the paragraph about study limitations, and I think they should. Also: who were the editors who did not respond? Do you have information about them that you could show in a table? If you can find and show a pattern of response (for example, editors from high-visibility journals would have less interest in responding?), then we can understand better this low response rate.

 6) **Figure 1.** Figure 1 improved a lot, thank you. Now it shows outliers and the types of interventions are clearer too (I wonder if you could put these “nicknames” in Box 1 too!).

 However, it is still a bit difficult to read/interpret. I believe the problem is that you chose to show **means** when you probably should have used **medians** (more appropriate to scores and to data that is not normally distributed). I suggest the authors try to check for data distribution, try to generate this figure with means, and that they also describe this method in the Methods – Data analysis section.

 8) The abstract improved, well done! However, the conclusions seem not to be conclusions at all, not based on your data. 

 The first sentence is your (and even mine!) opinion, but it not supported by your data. The second sentence tells about your aims, not your conclusions. 

 The same is valid for the conclusions at the end of the paper: they show valid comments/assumptions but nothing based on your data.

 Useful conclusions should be linked to your aims (to survey editors’ perceptions) and read like “the editors think that”... or “the perceptions of the editors are...”.

 18) **References**: you fixed most of them, but there are still references incomplete. Please review # 23, 24 and 28 and add the URLs.

**Additional concerns:**

 Regarding Data Analysis: I noticed you added text in this section about “excluding one questionnaire that was terminated early”. That called my attention: is this one of the 24 (and then we have 23 responded questionnaires) or was it a 25 ^th^ questionnaire? Also: how did you approach missing data? By excluding the participants? Was there any other case?

 Minor: Thank you for providing the names of the journals in Table 1. However, I suspect “Francis and Taylor” is not a journal name. Please, check (maybe there is a confusion with the publisher’s name “Taylor & Francis”, but what was the specific journal?).

I confirm that I have read this submission and believe that I have an appropriate level of expertise to confirm that it is of an acceptable scientific standard, however I have significant reservations, as outlined above.

# Response

**Published:** 2019-12-18

**Authors:** David Blanco

**Affiliations:** Universitat Politècnica de Catalunya, Spain

Thanks a lot, Patricia, for your extra comments and the time you took to make them. Please see below our response to them:

**Minor comments**

**References: you fixed most of them, but there are still references incomplete. Please review # 23, 24 and 28 and add the URLs.**

In our previous revised manuscript, we already had added the URLs for all Internet references. However, it seems that the copy-editors of F1000 did not explicitly show them and just include a link to them under “Reference source”.

**Regarding Data Analysis: I noticed you added text in this section about “excluding one questionnaire that was terminated early”. That called my attention: is this one of the 24 (and then we have 23 responded questionnaires) or was it a 25th questionnaire? Also: how did you approach missing data? By excluding the participants? Was there any other case?**

We excluded from analysis one questionnaire (the 25 ^th^) where the participant just opened the survey (and therefore SurveyMonkey registered it) but left without answering any question. For this reason, we decided not to count it as a participant. There were no further cases like this. We have made that more explicit in the text (first paragraph of Data analysis, first paragraph of Results).

**Minor: Thank you for providing the names of the journals in Table 1. However, I suspect “Francis and Taylor” is not a journal name. Please, check (maybe there is a confusion with the publisher’s name “Taylor & Francis”, but what was the specific journal?).**

We have checked that that was a mistake. Thanks to this, we have realised that for one of the existing journals we had two editors instead of one. We have subsequently adjusted the sample description.

**Major comments**

**2) Sample**. **The authors explained that they purposely searched for editors experienced with reporting guidelines. As much as I don’t agree with this approach, at least it was clearly stated now.**

**However, the explanation about the sample size as a study limitation still does not seem satisfactory. The authors added a comment in the discussion section attributing the sample size to a low response rate in surveys in general. But if the authors knew there was going to be a low response rate (and they cited very useful references for that), why did they stop recruiting? Why not try to approach other editors/journals? The authors should expand the explanation to respond to two questions:**

**What were the constraints in increasing the sample? Why did you stop recruiting?**

**Even choosing to use a “not representative sample”, can we trust that we should listen to the opinions of these 24 editors (a minority among the 99 invited) to say something is feasible or not, for example? What is the effect of this small sample size on the “take-home messages” of this study?**

**These points were not addressed in the paragraph about study limitations, and I think they should. Also: who were the editors who did not respond? Do you have information about them that you could show in a table? If you can find and show a pattern of response (for example, editors from high-visibility journals would have less interest in responding?), then we can understand better this low response rate.**

We have added further justifications on whether we believed that sample size and response rates were adequate for the purpose of the survey: “Despite having the option to increase the sample size by contacting more editors at a lower level of hierarchy in the journals we targeted, we decided not to do it based on the response rate of the survey. That approach would have changed our sampling frame and we would potentially have had less experienced editors commenting. We took that decision as the purpose of the survey was to tap into the experience of those who had tried interventions or had shown interest in this area, instead of seeking a representative sample of editors.”

We found no pattern of response among the journals/editors surveyed – participation was pretty homogeneous and covered journals of a variety of impact factors and areas, as can be seen in Table 1.

**6) Figure 1. Figure 1 improved a lot, thank you. Now it shows outliers and the types of interventions are clearer too (I wonder if you could put these “nicknames” in Box 1 too!).**

**However, it is still a bit difficult to read/interpret. I believe the problem is that you chose to show means when you probably should have used medians (more appropriate to scores and to data that is not normally distributed). I suggest the authors try to check for data distribution, try to generate this figure with means, and that they also describe this method in the Methods – Data analysis section.**

We are now reporting the results for the ease of implementation/effectiveness in terms of medians and quartiles instead of means and standard deviations. We have indicated this in the Methods-Data analysis section.

Regarding Figure 1, we have removed the means and we are now displaying the medians as blue horizontal lines. Moreover, we decided to remove the black points representing the individual scores as they do not allow us to see the medians in the graphs. In this way, Figure 1 is clearer and cleaner.   

**8) Conclusions. The abstract improved, well done! However, the conclusions seem not to be conclusions at all, not based on your data.**

**The first sentence is your (and even mine!) opinion, but it not supported by your data. The second sentence tells about your aims, not your conclusions.**

**The same is valid for the conclusions at the end of the paper: they show valid comments/assumptions but nothing based on your data.**

**Useful conclusions should be linked to your aims (to survey editors’ perceptions) and read like “the editors think that”... or “the perceptions of the editors are...”.**

We have modified the Conclusions both in the abstract and at the end of the paper, linking them to the survey aims (perceptions, barriers, facilitators…). We have also moved to the end of the Discussion section what was reported in the old Conclusions.

# Peer review: Reviewer response for version 2

**DOI:** 10.5256/f1000research.23906.r57748

**Published:** 2019-12-12

**Authors:** Dennis W. Lendrem

**Affiliations:** 1 Translational and Clinical Research Institute, Faculty of Medical Sciences, Newcastle University, Newcastle upon Tyne, UK; 2 NIHR Newcastle Biomedical Research Centre, Newcastle University and Newcastle upon Tyne Hospitals NHS Trust, Newcastle upon Tyne, UK

This paper tackles an important issue in science policy research. The authors have responded well to comments on an earlier draft. They have addressed the issues raised and the limitations of the study are clearly stated. While those limitations mean the survey results must be interpreted with caution, the findings are likely to inform discussions around the barriers to and facilitators of interventions to improve adherence to reporting guidelines.

I confirm that I have read this submission and believe that I have an appropriate level of expertise to confirm that it is of an acceptable scientific standard.

# Peer review: Reviewer response for version 1

**DOI:** 10.5256/f1000research.22597.r56388

**Published:** 2019-11-26

**Authors:** Patricia Logullo

**Affiliations:** 1 EQUATOR Network, University of Oxford, Oxford, UK

**Positive points:**

 This is a much-needed survey, and I am happy and thankful that the MiRoR team invested their time and expertise in undertaking it. The subject of the adoption of reporting guidelines in the editorial process is sensitive, relevant and worth exploring. Hearing editors is a crucial step for the development of interventions aimed to improve the quality of biomedical research reporting. I congratulate the authors for the initiative.

 The main result of the study (that the interventions perceived as effective are the most difficult to implement and vice-versa) points out to the need to verifying feasibility before proposing interventions, something that we should all think about.

 I hope the authors find these suggestions helpful and constructive, and that they find these to be positive contributions. 

**Major concerns: methodology**

**1)** **Sample characteristics.** Although the authors should be praised for the idea of asking editors about interventions to improve reporting, I have a concern about what editors should be surveyed. If one wants to explore the perceived ease of the implementation of one intervention, is it adequate to choose a population of editors whom we know are already “knowledgeable about the topic”?

 If these editors are aware of the reporting quality crisis and the reporting guidelines in general, wouldn’t these be more prone to adopt interventions that aim to improve the quality of publications? Therefore, aren’t these the people who would say “yes” to any intervention proposed?

 Should the authors not interview editors who have never heard about reporting guidelines too? Wouldn’t this heterogeneous sample be more representative of the scenario of difficulties — or the ease — of implementing reporting guidelines checks in the editorial process? Are the study results only applicable to journals that already endorse or require reporting guidelines?

 I would like to see comments about this limitation in the discussion if the authors agree with this or even if they do not.

**2)** **Sample size.** My second most important concern is about sample size. In the Methods section, the authors do not explain how they planned the sample size and based on what assumptions. 24% seems to be a very low response rate. Do the authors believe 24 is enough? This limitation should be discussed.

**3) Effectiveness.** When you asked your participants about their opinions on “how effective” something would be, what was the definition of effectiveness? Would it be their perception about the intervention improving the adherence to reporting guidelines checklists? Was it a more subjective view of “manuscript quality”? 

**4) Statreviewer.** The authors mention in the text, in Methods:

 “Based on feedback from the pilot we decided not to include the intervention “Implementation of the automatic tool Statreviewer14” since participants were not aware of this software and stated that their perceptions would strongly depend on details about how it operates which are not publicly available.”

 However, on page 7, Results, they state:

 “The implementation of reading tools that automatically assess adherence to RGs, such as Statreviewer14, were seen as potentially interesting interventions.”

 After all, was this intervention Statreviewer evaluated (as in Results) or was it not (as in Methods)? Do you mean they mentioned other tools, such as Penelope, GoodReports, Cobweb or others? Or were they referring to Statreviewer?

**Major concerns: reporting**

**5)** **Journals.** You interviewed 24 editors from 20 journals. What are these journals? I did not see a list of their names, and I believe it is important.

**6) Figure 1.** Figure 1 is difficult to read/interpret. I like the idea of having the two outcomes (ease of implementation and effectiveness) in one figure very much so that one can compare the two. However, the bar chart is not clear at all, nor easy to interpret. Colours do not help. The authors should consider building a figure with plot points (which would be easy, given the small sample) showing where each participant sits within the Likert scale — not bars.

 Another suggestion for Figure 1 (here, just a suggestion) is to add something to remind the readers what the interventions are. As Box 1 is far from the Figure, it is difficult to remember what each intervention is. It would be useful to have “nicknames”, for example:

 1. Page numbers

 2. Checklist

 3. Highlight

 4. Subheadings

 5. COBWEB

 6. Use RG

 7. Check RG

 8. Staff

 9. Training

**7) Peer reviewers’ checks.** The abstract is, in general, well written and clear. However, I got intrigued by this sentence:

 “Participants believed that checking adherence to guidelines goes beyond the role of peer reviewers and could decrease the overall quality of reviews.” 

 I was surprised that peer reviewers checking reporting guidelines adherence would decrease the overall quality of the paper. Then I reread it in the paper, but here put in a complete sentence, that makes sense:

 “Participants were concerned that the quality of peer review could be compromised as reviewers should focus on the manuscript’s content and not on the reporting issues.” 

 Meaning, I suppose, that peer reviewers would focus on checking reporting guidelines and not on their “expert view” of the study. I get it, but it is not very clear, neither in the text, nor in the abstract, and I invite the authors to rewrite both. Especially because “reporting guidelines” are about content too. They point out the content elements that should not be absent from papers, so using the word “content” in the manuscript text is not really appropriate. Please, re-evaluate.

**8)** As I said above, the abstract is good, and there is room for growth (you have now 232 words). However, I could not find the conclusions in the abstract helpful. Could you please inform better what you did conclude? What “points raised in the survey” should be considered?

**9)** **Checklists as forms.** ...“assessing completeness of reporting is a subjective task”. This is one of the most important sentences in this manuscript, it tells us about using reporting guidelines checklists as evaluation tools — for what they have not been created. I saw no discussion about this in your paper. 

**10)** **Discussion.** The discussion of the paper is short, and it lacks several points pointed above. Mainly, it does not discuss the limitations and potential bias of this study. Discussing limitations is a requirement from F1000Research journal.

**11)** You mention you did analyse qualitative information using **NVivo**. How did you do it?

**12)** There are at least eight items from the CHERRIES checklist that I could not see in your paper, and you might want to report, even if in a separate (or supplementary) box, from data protection, preventing multiple entries, to randomisation of items, and others.

**13) **From Box 1, I do not quite get the difference between Interventions 1 and 2. It seems that both ask authors to submit a populated/completed RG checklist. Is it that #1 asks for page numbers and #2 does not?

**14)** In my opinion, the sentences from “To contact editors...” to “improve adherence to RGs” belong more to the “Participants” section than to the “Procedure”. Especially the first sentence. 

 Also “Participants could suggest further editors that they considered could contribute to the survey” is something that I would consider as a snowballing or recruitment method, not the survey procedure per se. So, I would suggest putting everything related to recruitment together in one section, to make it easier for your reader (who might be interested in undertaking a similar survey) to find information on such a critical issue as recruitment. Also: how could participants suggest? Was it a specific question in the SurveyMonkey form?

**15)** Were participants given an estimation of the time required to answer the survey before they began?

**Minor: about citations**

**16) **Reference number 1 (EQUATOR Network website) is not a proper, complete reference. It points to the home page of the website, not to a document, and as so, it does not show from where the authors collected that information (the reader cannot find them in the home page). The correct way of citing would be adding the full URL. A suggestion: EQUATOR Network. What is a reporting guideline? Available at:

 http://www.equator-network.org/about-us/what-is-a-reporting-guideline/. Cited on 19/11/2019.

**17)** Something similar happens to reference number 13. Author and title are missing. It would help readers if it could be typed like this:

 Schoter S. Evidence-based publishing. BMJ. Available at: https://www.bmj.com/about-bmj/evidence-based-publishing. Cited on 19/11/2019.

**18) **Reference 14 also lacks authors and titles. References 13, 14, 16, 21 and 22 lack a lot of elements necessary for readers to be able to find them. When working in a printed version (as I am now), you cannot see what the document is, where it is published or by whom — these are available only by those reading online and able to “click” on the links. 

**19)** I would change the sentence:

 “Raw survey results are given as Underlying data ^20^.”

 To something clearer and that does not require the reader to go to the reference list to understand what it is. 

 “Table XX in the supplementary file X ^20^ shows the responses from all 24 participants.” 

**20)** Has the study by Hawwash *et al.* (reference 25) been published yet? (The authors cited the protocol only). Do the authors plan to cite other electronic tools that are out there?

 Thank you for the opportunity of reading and evaluating this paper.

I confirm that I have read this submission and believe that I have an appropriate level of expertise to confirm that it is of an acceptable scientific standard, however I have significant reservations, as outlined above.

# Response

**Published:** 2019-12-09

**Authors:** David Blanco

**Affiliations:** Universitat Politècnica de Catalunya, Spain

We thank you for your time and detailed comments on the manuscript. Please find below a point by point response to them:

**Major concerns: methodology**

**1) Sample characteristics. Although the authors should be praised for the idea of asking editors about interventions to improve reporting, I have a concern about what editors should be surveyed. If one wants to explore the perceived ease of the implementation of one intervention, is it adequate to choose a population of editors whom we know are already “knowledgeable about the topic”?**

**If these editors are aware of the reporting quality crisis and the reporting guidelines in general, wouldn’t these be more prone to adopt interventions that aim to improve the quality of publications? Therefore, aren’t these the people who would say “yes” to any intervention proposed?**

**Should the authors not interview editors who have never heard about reporting guidelines too? Wouldn’t this heterogeneous sample be more representative of the scenario of difficulties — or the ease — of implementing reporting guidelines checks in the editorial process? Are the study results only applicable to journals that already endorse or require reporting guidelines?**

**I would like to see comments about this limitation in the discussion if the authors agree with this or even if they do not.**

Firstly, we have now included some more information in the “Participants” section to make it clear what kind of participants we were aiming to reach. Secondly, we have discussed in the Discussion section the other points you mention.

**2) Sample size. My second most important concern is about sample size. In the Methods section, the authors do not explain how they planned the sample size and based on what assumptions. 24% seems to be a very low response rate. Do the authors believe 24 is enough? This limitation should be discussed.**

We have now discussed these points in the Discussion section.

**3) Effectiveness. When you asked your participants about their opinions on “how effective” something would be, what was the definition of effectiveness? Would it be their perception about the intervention improving the adherence to reporting guidelines checklists? Was it a more subjective view of “manuscript quality”?**

Thanks to your comment, we have just realised that we only mentioned that we were talking about “effectiveness at improving adherence to RGs” in the methods section, but not in the abstract and introduction. We have now clarified that in the abstract and introduction, and also in some parts of the methods, results and discussion to remind the readers that we refer to effectiveness at improving adherence to RGs.

**4) Statreviewer. The authors mention in the text, in Methods:**

**“Based on feedback from the pilot we decided not to include the intervention “Implementation of the automatic tool Statreviewer14” since participants were not aware of this software and stated that their perceptions would strongly depend on details about how it operates which are not publicly available.”**

**However, on page 7, Results, they state:**

**“The implementation of reading tools that automatically assess adherence to RGs, such as Statreviewer14, were seen as potentially interesting interventions.”**

**After all, was this intervention Statreviewer evaluated (as in Results) or was it not (as in Methods)? Do you mean they mentioned other tools, such as Penelope, GoodReports, Cobweb or others? Or were they referring to Statreviewer?**

We have clarified in the results section (“Further interventions and incentives for authors and journals”) that Statreviewer was not included in the survey but mentioned by some participants when asked about further potentially effective interventions.

**Major concerns: reporting**

**5) Journals. You interviewed 24 editors from 20 journals. What are these journals? I did not see a list of their names, and I believe it is important.**

The 20 journals represented are now listed in Table 1. We have also improved the description of the sample in the first paragraph of the Results section.

**6) Figure 1. Figure 1 is difficult to read/interpret. I like the idea of having the two outcomes (ease of implementation and effectiveness) in one figure very much so that one can compare the two. However, the bar chart is not clear at all, nor easy to interpret. Colours do not help. The authors should consider building a figure with plot points (which would be easy, given the small sample) showing where each participant sits within the Likert scale — not bars.**

We have substituted the bar plot by a box plot. We have also plotted the individual and mean scores for each intervention and each outcome. Everything is explained in the legend.  

**Another suggestion for Figure 1 (here, just a suggestion) is to add something to remind the readers what the interventions are. As Box 1 is far from the Figure, it is difficult to remember what each intervention is. It would be useful to have “nicknames”, for example:**

**1. Page numbers**

**2. Checklist**

**3. Highlight**

**4. Subheadings**

**5. COBWEB**

**6. Use RG**

**7. Check RG**

**8. Staff**

**9. Training**

We have incorporated similar nicknames to help readers remember the interventions.

**7) Peer reviewers’ checks. The abstract is, in general, well written and clear. However, I got intrigued by this sentence:**

**“Participants believed that checking adherence to guidelines goes beyond the role of peer reviewers and could decrease the overall quality of reviews.”**

**I was surprised that peer reviewers checking reporting guidelines adherence would decrease the overall quality of the paper. Then I reread it in the paper, but here put in a complete sentence, that makes sense:**

**“Participants were concerned that the quality of peer review could be compromised as reviewers should focus on the manuscript’s content and not on the reporting issues.”**

**Meaning, I suppose, that peer reviewers would focus on checking reporting guidelines and not on their “expert view” of the study. I get it, but it is not very clear, neither in the text, nor in the abstract, and I invite the authors to rewrite both. Especially because “reporting guidelines” are about content too. They point out the content elements that should not be absent from papers, so using the word “content” in the manuscript text is not really appropriate. Please, re-evaluate.**

We completely agree with the point you raise. We have re-written this point in the Abstract, Results and Conclusions. “Participants believed that checking adherence to guidelines goes beyond the role of peer reviewers and were concerned that the quality of peer review could be compromised. Reviewers are generally not expected to focus on reporting issues but on providing an expert view on the importance, novelty, and relevance of the manuscript.”

**8) As I said above, the abstract is good, and there is room for growth (you have now 232 words). However, I could not find the conclusions in the abstract helpful. Could you please inform better what you did conclude? What “points raised in the survey” should be considered?**

We have re-written and expanded the Conclusions in the abstract. The abstract is now 295 words.

**9) Checklists as forms. ...“assessing completeness of reporting is a subjective task”. This is one of the most important sentences in this manuscript, it tells us about using reporting guidelines checklists as evaluation tools — for what they have not been created. I saw no discussion about this in your paper.**

We have now discussed this point in the paragraph before the limitations of the study.

**10) Discussion. The discussion of the paper is short, and it lacks several points pointed above. Mainly, it does not discuss the limitations and potential bias of this study. Discussing limitations is a requirement from F1000Research journal.**

We have now included such a section in the Discussion section.

**11) You mention you did analyse qualitative information using NVivo. How did you do it?**

We have now provided further information on how we did the qualitative analysis with NVivo (“Data analysis” section).

**12) There are at least eight items from the CHERRIES checklist that I could not see in your paper, and you might want to report, even if in a separate (or supplementary) box, from data protection, preventing multiple entries, to randomisation of items, and others.**

We have now included the information corresponding to the missing items throughout the whole Methods section.

**13) From Box 1, I do not quite get the difference between Interventions 1 and 2. It seems that both ask authors to submit a populated/completed RG checklist. Is it that #1 asks for page numbers and #2 does not?**

#1 asks for page numbers and #2 for text from the manuscript. Some people may think that if you nudge authors to have to copy-paste text into the checklist they may tend to take it more seriously than if you just ask them to put a page number. We have slightly modified their wording in Box 1 to make it clearer.

**14) In my opinion, the sentences from “To contact editors...” to “improve adherence to RGs” belong more to the “Participants” section than to the “Procedure”. Especially the first sentence. Also “Participants could suggest further editors that they considered could contribute to the survey” is something that I would consider as a snowballing or recruitment method, not the survey procedure per se. So, I would suggest putting everything related to recruitment together in one section, to make it easier for your reader (who might be interested in undertaking a similar survey) to find information on such a critical issue as recruitment. Also: how could participants suggest? Was it a specific question in the SurveyMonkey form?**

We have restructured that part of the manuscript and divided it into “Recruitment” and “Survey administration”. We have also provided the information requested.

**15) Were participants given an estimation of the time required to answer the survey before they began?**

We have now included this information (“Survey administration” subsection).

**Minor: about citations**

**16) Reference number 1 (EQUATOR Network website) is not a proper, complete reference. It points to the home page of the website, not to a document, and as so, it does not show from where the authors collected that information (the reader cannot find them in the home page). The correct way of citing would be adding the full URL. A suggestion: EQUATOR Network. What is a reporting guideline? Available at:**

**http://www.equator-network.org/about-us/what-is-a-reporting-guideline/. Cited on 19/11/2019.**

Done.

**17) Something similar happens to reference number 13. Author and title are missing. It would help readers if it could be typed like this:**

**Schoter S. Evidence-based publishing. BMJ. Available at: https://www.bmj.com/about-bmj/evidence-based-publishing. Cited on 19/11/2019.**

Done.

**18) Reference 14 also lacks authors and titles. References 13, 14, 16, 21 and 22 lack a lot of elements necessary for readers to be able to find them. When working in a printed version (as I am now), you cannot see what the document is, where it is published or by whom — these are available only by those reading online and able to “click” on the links.**

Done.

**19) I would change the sentence:**

**“Raw survey results are given as Underlying data20.”**

**To something clearer and that does not require the reader to go to the reference list to understand what it is.**

**“Table XX in the supplementary file X20 shows the responses from all 24 participants.”**

Done.

**20) Has the study by Hawwash et al. (reference 25) been published yet? (The authors cited the protocol only). Do the authors plan to cite other electronic tools that are out there?**

We have now cited the results paper by Hawwash et al., which got published on 6 November. Regarding the electronic tools, we have already cited COBWEB and Statreviewer. We did not cite Penelope as it does not explicitly check RG requirements – it just helps authors submit the relevant RG checklist.

# Peer review: Reviewer response for version 1

**DOI:** 10.5256/f1000research.22597.r55889

**Published:** 2019-11-20

**Authors:** Gerard Urrutia

**Affiliations:** 1 Iberoamerican Cochrane Center, Sant Pau Biomedical Research Institute, Barcelona, Spain

This is an interesting and original study that deepens the knowledge about interventions aimed at improving adherence to reporting guidelines by medical journals, an important issue. The study focuses on the perspective of the biomedical editors and provides valuable information on their perceptions of various interventions that have been proposed, identifies barriers and facilitators and provides possible solutions.

 However, the study includes a limited sample of participants (24 editors belonging to 20 medical journals), which limits the scope of the conclusions. Although the study, according to its stated objectives, does not intend to offer a representative view of the editors of all medical journals, the limited number of participants as well as their representativeness (the sources from which they were identified and selected allows us to think that it is a highly selected sample of publishers with previous experience and a potential motivation in the subject), is not mentioned in the discussion. It is noteworthy that, despite this potentially "favourable" sample, the response rate is very low (24%). What would have been the participation rate and the responses if a larger and more representative sample had been selected? Although this was not an objective of the study, it would deserve some reflection in the discussion.

 It would also be useful to provide details on the origin of the participants in the survey according to the sources of selection, which would improve the description of the sample.

 The results presented both in the section on current practices as well as in perceptions and in barriers and facilitators are consistent, clear and practical. The analysis is descriptive and does not pose difficulties.

I confirm that I have read this submission and believe that I have an appropriate level of expertise to confirm that it is of an acceptable scientific standard.

# Response

**Published:** 2019-12-09

**Authors:** David Blanco

**Affiliations:** Universitat Politècnica de Catalunya, Spain

We thank you for your time and comments on the manuscript. Please find below a point by point response to them:

**However, the study includes a limited sample of participants (24 editors belonging to 20 medical journals), which limits the scope of the conclusions. Although the study, according to its stated objectives, does not intend to offer a representative view of the editors of all medical journals, the limited number of participants as well as their representativeness (the sources from which they were identified and selected allows us to think that it is a highly selected sample of publishers with previous experience and a potential motivation in the subject), is not mentioned in the discussion.**

 We have now discussed these points in the Discussion section.

**It is noteworthy that, despite this potentially "favourable" sample, the response rate is very low (24%). What would have been the participation rate and the responses if a larger and more representative sample had been selected? Although this was not an objective of the study, it would deserve some reflection in the discussion.**

 We have now included some comments about the response rate in a new paragraph on Limitations (Discussion section).

**It would also be useful to provide details on the origin of the participants in the survey according to the sources of selection, which would improve the description of the sample.**

 We have now added that information to the first paragraph of the Results section.

# Peer review: Reviewer response for version 1

**DOI:** 10.5256/f1000research.22597.r55890

**Published:** 2019-11-11

**Authors:** Dennis W. Lendrem

**Affiliations:** 1 Translational and Clinical Research Institute, Faculty of Medical Sciences, Newcastle University, Newcastle upon Tyne, UK; 2 NIHR Newcastle Biomedical Research Centre, Newcastle University and Newcastle upon Tyne Hospitals NHS Trust, Newcastle upon Tyne, UK

This paper reports on the views of a sample of biomedical editors on editorial interventions to improve adherence to reporting guidelines. The paper is clear, transparent, and well documented.

 A limitation of the study is that the results are based upon a survey return rate of just 24%. The returns are likely biased favouring editors with stronger views on reporting guidelines. In addition, it is not clear whether all ten of the top-ten journals were represented, or whether one or more journals dominate the response rate. In addition it is not completely clear how many participants came from the MiRoR Network, or journals previously publishing studies on Reporting Guidelines.

 Figure 1 is hard work to interpret and might be presented more usefully as two separate graphs. In addition, the colour scheme could be changed to highlight those interventions considered "Very easy" or "Moderately easy" to implement, and those ranked as "Very effective" or "Moderately effective". (Note that the proportions of each score could still be retained.) 

 However, this is a useful addition to the literature offering valuable insight into some views on guidelines and candidate interventions promoting closer adherence to guidelines. The paper would be improved by a paragraph on Limitations of the Study.

I confirm that I have read this submission and believe that I have an appropriate level of expertise to confirm that it is of an acceptable scientific standard, however I have significant reservations, as outlined above.

# Response

**Published:** 2019-12-09

**Authors:** David Blanco

**Affiliations:** Universitat Politècnica de Catalunya, Spain

We thank you for your time and comments on the manuscript. Please find below a point by point response to them:

**A limitation of the study is that the results are based upon a survey return rate of just 24%. The returns are likely biased favouring editors with stronger views on reporting guidelines.**

We have now reflected on this point in the Discussion section.

**In addition, it is not clear whether all ten of the top-ten journals were represented, or whether one or more journals dominate the response rate. In addition it is not completely clear how many participants came from the MiRoR Network, or journals previously publishing studies on Reporting Guidelines.**

We have now added that information to the first paragraph of the Results section.

**Figure 1 is hard work to interpret and might be presented more usefully as two separate graphs. In addition, the colour scheme could be changed to highlight those interventions considered "Very easy" or "Moderately easy" to implement, and those ranked as "Very effective" or "Moderately effective". (Note that the proportions of each score could still be retained.)**

In order to make Figure 1 easier to interpret, we have substituted the bar plot by a box plot. We have also plotted the individual and mean scores for each intervention and each outcome. Everything is explained in the legend.  

However, we prefer not to separate it into two graphs as we believe it is interesting for the readers to be able to compare the scores that each intervention obtains for each of the two outcomes.

**The paper would be improved by a paragraph on Limitations of the Study.**

We have now included a section on Limitations of the study in the Discussion section.
